# Conservation and exploitation status of skate species (Batoidea: Rajidae) in the Balearic Islands, western Mediterranean

**DOI:** 10.1371/journal.pone.0347768

**Published:** 2026-04-29

**Authors:** Francesca Ferragut-Perello, Sergio Ramírez-Amaro, Natalia Petit-Marty, M. Teresa Farriols, Antoni Quetglas, Beatriz Guijarro, Francesc Ordines

**Affiliations:** 1 Centre Oceanogràfic de les Balears (COB-IEO), CSIC, Palma, Spain; 2 Laboratori de Genètica, Universitat de les Illes Balears, Palma, Spain; 3 Instituto de Investigacions Mariñas (IIM), CSIC, Vigo, Spain; University of Messina, ITALY

## Abstract

Batoids play key roles in marine ecosystems, yet their slow life history traits make them particularly vulnerable to overexploitation. The Balearic Islands remain a hotspot of batoid diversity, although multiple species could face conservation concerns. We combine genetic analyses based on mitochondrial COI genetic diversity with data-poor stock assessment methodologies to assess the conservation and exploitation status of the most abundant Rajidae species in the area. A total of 181 sequences of five wide distributed species and two Mediterranean endemic species (*Raja radula* and *Raja polystigma),* were analysed and compared against a Mediterranean–Atlantic Rajidae genetic diversity framework. We also assessed the stocks of *Dipturus oxyrinchus* and *Raja clavata*, using the Bayesian state-space implementation of the Schaefer production model, as well as *R. polystigma*, using a length-based Bayesian biomass estimation method. The nucleotide diversity of most species was below the lower confidence interval of the median of the comparative framework. Despite this, the assessed species showed signs of recovery and sustainable exploitation. The biomass of *R. clavata* and *D. oxyrinchus* showed an important increase in recent years following reductions in fishing effort, while *R. polystigma* showed stability and sustainable exploitation. *Raja brachyura*, *R. radula* and *Leucoraja naevus*, with low overlap between their bathymetric distribution and the bottoms most intensively exploited by the bottom trawl fleet, showed the highest genetic diversities. Similarly, the low overlap of this fishery, at least with important fractions of the populations of *R. clavata*, *R. polystigma* and *D. oxyrinchus*, is in coincidence with their resilience to fishing exploitation. These findings highlight the importance of depth distribution in shaping resilience, emphasizing the need for species- and region-specific conservation strategies for these vulnerable species. In this sense, the integration of genetic monitoring with stock assessments is gaining relevance for detecting hidden vulnerabilities on threatened species such as batoids.

## Introduction

Batoidea group (skates, rays and allies) comprises more than half of species diversity among the chondrichthyan fishes [1]. They usually occupy a position of top or meso-predators in the ecosystems and, consequently, have a crucial role maintaining structure and functioning of food webs. The decrease of their populations can cause trophic cascades through top-down effects, and thus notably modify marine communities and ecosystems [2]. Like sharks, batoids are typical slow living strategists [3], characterised by slow growth, low fecundity, and late maturity. This makes batoid populations especially vulnerable to overexploitation [1,4,5], which currently constitutes its main threat [6]. This vulnerability highlights the urgent need to ensure their conservation, since reduced biodiversity affects the functioning of ecosystems and significantly decreases the services that they provide [7]. Although batoids, and chondrichthyans in general, are mainly caught as a bycatch, they are almost entirely retained [6].

The current status of Batoidea worldwide, according to the International Union for the Conservation of Nature [8], shows that around 30% of the 1234 species assessed are threatened, with many species undergoing population declines, and even local extinctions [1]. The situation is particularly alarming in the Mediterranean, where at least 42% of the Batoidea (16 of 38 species) face an elevated extinction risk [9]. In the Balearic Islands, for instance, where species such as *Glaucostegus cemiculus and Torpedo torpedo* were once common, they are now catalogued as locally extinct [10]. Despite this, the area still represents a hotspot of biodiversity and abundance of Batoidea in the western Mediterranean [11–14], with a particularly high biodiversity of the Rajidae family [13,15]. This high biodiversity has been related to the historically lower fishing effort in the Balearic Islands compared to adjacent areas such as the Iberian Peninsula, which results in a healthier state of exploitation of demersal resources [16].

The main commercial fisheries in the Balearic Islands are the bottom trawl and the small-scale fleets [17]. Whereas the small-scale fishery (SSF) vessels represent 80% of the fishing fleet, the bottom otter trawl fishery (OTB) concentrates more than 70% of the commercial landings [16]. Both fleets have shown reductions of up to 50% in the number of vessels since the last third of the twentieth century [18]. In the case of the OTB, such reductions have implied a 60% decrease in the number of fishing days during the last 20 years [19]. Moreover, by 2024, the implementation of the multiannual plan for the fisheries exploiting demersal stocks in the western Mediterranean Sea (EU-MAP) [20] had resulted in an additional reduction of up to 40% of the fishing days of the OTB relative to the 2015–2017 period. This EU-MAP is primarily based on a bottom trawl fishing effort regime, initiated in 2020, which systematically reduces annual fishing days as a percentage of that 2015–2017 baseline. Complementing this effort control are technical conservation measures, including spatio-temporal closures to protect juvenile and spawning aggregations of demersal stocks and modifications to trawl gear to improve selectivity. Furthermore, annual catch limits were established for key commercial species such as the blue and red shrimp (*Aristeus antennatus*).

One of the most abundant batoid species inhabiting the waters off the Balearic Islands, the skate *Raja radula*, a Mediterranean endemism, is listed as Endangered by the IUCN Red List of Species [21], whereas *Dipturus oxyrinchus*, *Raja clavata*, *Leucoraja naevus* and *Raja brachyura* are catalogued as Near Threatened [22–25]. By contrast, *Raja miraletus* and *Raja polystigma,* another Mediterranean endemism, are considered species of Least Concern [26,27]). Although several of these skates (e.g., *R. clavata*, *D. oxyrinchus*, *R. miraletus*, *L. naevus* and *R. polystigma*) are frequent bycatches of the Mediterranean OTB [11,14,28,29], most of them are discarded [28,30]. Species inhabiting shallow waters, such as *R. radula* and *R. brachyura*, are mainly caught by the SSF [31,32]. In the Balearic Islands, most batoid species are discarded due to small individual size, with the exception of *D. oxyrinchus* and *R. clavata*, this last one representing up to 83% of the landings of the skate commercial category [33].

Determining the exploitation and conservation status of by-catch species, such as batoids, can be difficult due to limited data availability. In these cases, several assessment tools for data-poor stocks have traditionally been used, such as surplus production models based on catch data and an index of biomass [34], as well as methods based on length frequency data [35]. More recently, a novel approach based on genetic diversity in the mitochondrial gene of Cytochrome C Oxidase subunit I (COI; DNA barcode) has been proposed as a diagnostic for estimating the conservation status of marine species [36]. This approach was later validated for exploited teleost fishes [37], confirming that COI diversity aligns with expectations and theoretical predictions tied to commercial exploitation, life-history traits, and stock assessment results.

In the present work, we apply a multidisciplinary approach. This involves two methods: *i)* an innovative method based on mitochondrial COI genetic diversity to determine the conservation status of the populations of the most abundant Rajidae species, and *ii)* data-poor stock assessment models, to evaluate the effect of the drastic decrease of commercial fishing effort observed in the Balearic Islands over the last decades, and especially after 5 years of the implementation of the EU-MAP [20], on their exploitation state.

## Materials and methods

### Study area and sample sources

**Fig 1:**
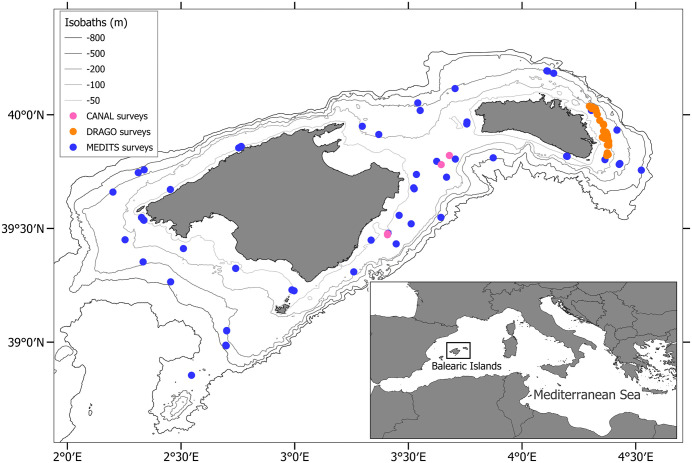
Map of the study area (Balearic Islands, western Mediterranean) showing the locations from where the analysed samples were collected during three different scientific surveys.

The data and samples used in the present work were obtained from two different sources: scientific surveys carried out off the Balearic Islands (Fig 1) and daily sale bills from Mallorca fish auction wharf, supplied by the local fish producers organisation. Most data and samples were collected during the Mediterranean bottom trawl surveys (MEDITS; [38,39]) performed between 2001 and 2024. MEDITS surveys co-funded by the European Union through the European Maritime and Fisheries Fund (EMFF), within the National Programme of collection, management and use of data in the fisheries sector and support for scientific advice regarding the Common Fisheries Policy (PNDB). These surveys take place annually during spring-summer on bottom trawl fishing grounds from 50 to 800 m depth around the islands of Mallorca and Menorca. The sampling scheme followed a standardized protocol [38,39] approved by international authorities (EU/DG Mare, FAO/GFCM). If a live specimen of a rare species or a species subject to conservation measures were caught, it was quickly sampled (4–5 min) and returned to the sea unharmed, giving it a chance of survival, following recommendation GFCM/36/2012/3 (https://www.fao.org/gfcm/decisions/en/ accessed on January 11 2025) on fisheries management measures for conservation of sharks and rays in the GFCM area. During the hauls, horizontal and vertical openings of the net (GOC-73) are recorded using an acoustic MARPORT system. In each sample haul, the catch was sorted out by species. Then, their abundance and biomass, as well as length frequency distribution of fish was determined. Abundances were standardized to 1 km^2^ dividing the number of individuals by the area sampled in each haul, which is estimated from the distance covered and the horizontal opening of the net. Then, the average standardized abundance was multiplied by the area (km^2^) of every depth stratum. For genetic analysis, we collected small pieces (1 cm^2^ per individual) of pelvic fin tissue from individuals of the following species: *Dipturus oxyrinchus, Leucoraja naevus, Raja brachyura, Raja miraletus, Raja polystigma* and *Raja radula*. Tissues were preserved in 96% ethanol and stored at −20°C. The samples for genetic analysis were collected during the MEDITS surveys from 2013 to 2022. Additionally, we completed samples collecting tissue during the DRAGO and CANAL bottom trawl surveys carried out in 2019 and 2022, respectively (Fig 1). The DRAGO survey was a controlled scientific experiment carried out on the continental shelf of Menorca in September 2019. Its primary objective was to test the effectiveness of a modified bottom trawl groundrope in reducing benthic discards within sensitive maërl beds. Using two commercial vessels fishing in parallel, the survey compared commercial yields, total catches, and discard compositions between the experimental gear and the traditional trawl net employed by the local fleet. The CANAL survey aimed to assess the effects of bottom trawl protection on the trophic relationships of benthophagous species. Using a standardized GOC-73 experimental trawl, 23 hauls were performed between 58–75 m depth to compare three distinct areas: a trawling-prohibited zone, a managed area with low pressure, and an adjacent fished area.

### Conservation status

The DNA from tissue samples was extracted with the DNeasy Blood and Tissue Extraction kit (Qiagen, West Sussex, UK). Polymerase Chain Reaction (PCR) was used to amplify the partial mitochondrial gen Cytochrome C Oxidase subunit I (COI; DNA barcode), with universal Fish primers FF2d/FR1d [40]. The PCR conditions were: one cycle of initial denaturation of 2 min at 95 °C; 35 PCR cycles of 1 min at 94 °C, 1 min at 54 °C and 1 min at 72 °C; and one cycle of final extension at 72°C for 10 min. The PCR products were purified using QIAquick® PCR Purification Kit (QUIAGEN) and their sequencing by the Sanger method was outsourced. Then, sequences were edited and aligned with BioEdit 7.0.5.2 software [41], and deposited in the GenBank database (http://www.ncbi.nlm.nih.gov/genbank/ accessed on March 21 2025) (Table 1). We also added additional COI sequences to the alignments, previously obtained by the authors of the present work for these species in the Balearic Islands.

**Table 1:** GenBank IDs of the sequences used for the genetic diversity analysis of different Rajidae species from the Balearic Sea (western Mediterranean). The number of samples (N) is also shown.

Species	N	GenBank IDs
*Dipturus oxyrinchus*	1	KY949111
	2	KU761956 - KU761957
	26	PV524769 - PV524794
*Leucoraja naevus*	5	KY949101 - KY949105
	22	PV524795 - PV524816
*Raja brachyura*	5	KY949096 - KY949100
	26	PV524817 - PV524842
*Raja miraletus*	3	KY949085 - KY949087
	26	PV524843 - PV524868
*Raja polystigma*	14	KY949056 - KY949057; KY949059 - KY949070
	23	PV524869 - PV524891
*Raja radula*	5	KY949096 - KY949100
	23	PV524892 - PV524914

Genetic diversity indices were estimated with the dataset of each species using Arlequin 3.1 [42]: number of haplotypes (N_H_), haplotype diversity ($h=\frac{n}{n-1}(1-\sum_{i}{p_{i}}^{2})$; where *p*_*i*_ is the frequency of the *i*th haplotype and *n* is the number of sequences [43], and nucleotide diversity ($\pi =\frac{n}{n-1}\sum_{ij}x_{i}x_{j}\pi_{ij})$; where *x*_*i*_ and *x*_*j*_ are the frequencies of the *i*th and *j*th sequences, *π*_*ij*_ is the number of nucleotide differences per nucleotide site between the *i*th and *j*th sequences, and *n* is the number of sequences [43].

We created a comparative framework of genetic diversity based on COI for the Rajidae populations throughout the Mediterranean Sea and the Atlantic Ocean. To do so, we created a dataset of COI genetic diversity indices based on published information and available sequences in GenBank, considering exclusively areas or studies with more than 10 sequences. When a study reported diversity values for multiple areas, we treated them as separate populations only if COI-base population differentiation had been detected; differentiation based on other genetic markers was not considered. In cases where no COI-based differentiation was found between geographically defined subareas, we recalculated the diversity indices by merging these subunits into a broader geographic region, i.e., combining adjacent areas into a coherent region as defined by the reported COI population structure (e.g., the Sicilian Strait and the West Ioanian Sea or East and West Sardinia). This process involved adding the number of sequences, determining the number of haplotypes based on the haplotype networks provided in the particular study, and averaging the remaining statistics. For studies that did not report genetic diversity indexes but provided at least 10 COI sequences for a Rajidae species population, we calculated them following the same methodology described above for the Balearic Islands populations. Once the genetic dataset was created, bootstrap resampling was applied to estimate the median nucleotide diversity (π) values from the COI gene. We used the median as a measure of central tendency because of the non-parametric distribution of our data, following the approach of Petit-Marty et al. [37]. Using the *Boot* package in R software [44], 10000 bootstrap replicates were generated by random sampling with replacement from the original dataset, maintaining the original sample size for each replicate. Then, the percentile bootstrap procedure was used to calculate the median value of π and its 95% confidence intervals (95%CI). This method was performed separately for *i)* the complete dataset (including both Atlantic and Mediterranean populations’ information), *ii)* the subset containing only information from the Atlantic populations, and *iii)* the subset containing only Mediterranean populations’ information. The median and its 95%CI value from the complete dataset were used as nucleotide diversity comparative framework. We considered a population to be in a worse conservation status than the average of those included in the comparative framework if its nucleotide diversity fell below the lower boundary of the framework’s median 95%CI.

Additionally, haplotype networks were constructed for each of the studied *Rajidae* species in the Balearic Islands to determine whether populations possess unique haplotypes or share them with other populations. In cases of shared haplotypes, the networks also allowed us to assess the extent and geographic pattern of this sharing. For this, we gathered all the different haplotypes of the comparative framework dataset, and analysed sharedness of them among the considered regions, using the PopART software, applying the median joining network method [45]. However, as most of the referenced studies in the comparative framework provided sequences only for distinct haplotypes (without including their frequencies) the resulting networks reflect the presence and distribution of haplotypes across the different considered populations, but not their relative abundance. For this reason, we do not present the networks’ figures but instead report the qualitative information on which haplotypes are shared or unique among populations.

### Exploitation status

We assessed the exploitation status for *D. oxyrinchus, R. clavata* and *R. polystigma*. These species were selected according to the data availability that allowed the application of specific stock assessment models. Thus, for the first two species, long time series of commercial catches were available, but for *R. polystigma*, such data were lacking. Instead, catches of this species during the MEDITS surveys were sufficient to determine the population structure. Therefore, the Bayesian state-space implementation of the Schaefer production model (BSM) was used for *D. oxyrinchus* and *R. clavata* and the length-based Bayesian biomass estimation method (LBB) was applied to *R. polystigma*. R routines [46] to perform both models can be found at the open-source Catch Length Abundance (CLA) stock assessment toolbox at GitHub (https://github.com/ktouloum/CLA_stock_assessment/ accessed on March 12 2025) [47].

The BSM is fitted to catch and biomass or catch-per-unit-of-effort (CPUE) data and estimates biomass, exploitation rate, MSY and related ﬁsheries reference points from catch data and resilience of the assessed species [48]. Catch data of *R. clavata* since 1964 were obtained from a reconstructed landings time series from Palma’s fish auction wharf. This series is based on the reconstruction by Ferragut-Perello et al. [33], which we updated for this work by applying their calculated proportion of *R. clavata* within the skate commercial category to new commercial landings data from 2022 to 2024. The original proportion was estimated from observer data on board the commercial fleet of Mallorca and refers to the skate category that includes most Rajidae species (mainly *Raja* spp. and *Leucoraja* spp.), though non-*R. clavata* species are usually not included, as they are discarded due to their smaller size [33]. By contrast, the presence of a specific commercial category for *D. oxyrinchus* since 2004 allowed to obtain a time series of landings of this species for the last 20 years.

The CPUE time series was calculated using the annual fishing effort since 1964 (restricted from 2004 for *D. oxyrinchus*), obtained as the actual yearly engine horse power of the bottom trawlers operating in the area, as skate species assessed are caught almost entirely by this fleet [49]. Communications with fishermen enabled the use of actual, rather than nominal (declared), engine power (in HP) for each vessel, including changes in engine power and the corresponding increase in HP throughout the entire time series (1964–2024). These variations in gear power were associated with increases in gear size, vertical net opening, and trawling speed. Changes in the permitted time at sea, resulting from successive adjustments to fishing regulations over the time series, were also considered—both in terms of the number of fishing days per week and hours per day. Accounting for temporal variations in both gear power and fishing time allowed for the inclusion of technological creep effects in the estimation of fishing effort [49]. Other components of technological creep, such as improvements in navigation systems and gear efficiency beyond those associated with engine power, were not explicitly accounted for in this standardization. Monthly fishing effort was calculated as the sum of the horsepower of all active vessels during a given month, weighted by the permitted time at sea. Annual fishing effort was subsequently derived from the sum of monthly estimates. Then, CPUE was calculated as catch (landings) divided by this effort (Annual HP). To assess potential spatial shifts in fishing effort of the bottom trawl fleet following the drastic reduction in total effort over the last decade, an analysis of fishing days by bathymetric stratum (continental shelf, 50–100 m of depth; shelf-break, 100–200 m; upper slope, 200–500 m; and middle slope, 500–800 m) was conducted using the data collected through the Vessel Monitoring System (VMS) in order to get effort measures by depth strata. The VMS data consists on registers of geographical position, date, time, and instant velocity of each vessel, approximately every two hours [31]. For the present analyses, we used up to 4,320,000 VMS signals emitted between 2011 and 2024. In order to take only into account signals emitted when the fleet is fishing, we only considered those signals emitted when the boat was in the fishing grounds and sailing at a velocity within the range used by trawlers during the fishing activity in the Balearic Islands, between 2 and 3.6 kn (around 480,000 signals). Later, each signal was assigned to a depth stratum using a Geographic Information System (GIS) in order to calculate the fishing effort in terms of fishing days per strata. In the Balearic Islands, boats are allowed to fish only 12 hours per day (daily fishing trips). However, it is common practice in the area that a boat exploits different depth strata targeting different resources. One of the most common mixed strategies is a fishing trip performing a first haul on the continental shelf targeting stripped red mullet, squid, octopus, etc., followed by one in the middle slope targeting blue and red shrimp [16]. Hence, in the case of a boat emitting signals from 2 or three depth strata, only one half or third of a fishing day was considered in each stratum, respectively. Linear regression was performed to analyze trends in fishing days, both for the total time series and for each bathymetric stratum separately.

The BSM requires a resilience (r) input prior, which was set at 0.05–0.5 according to the life-history traits of both species [48,50]. The model also needs priors of the relative biomass compared to the carrying capacity (B/k) at different points, at least at the start, of the time series. We used two B/k priors for *R. clavata*, one for the initial year (1964), when the industrial fishing in the area had very recently started [49], with B/k ranging from 0.62 to 0.96, corresponding to an unexploited to low exploited stock with high biomass, and another for the year 2000, when the bottom trawl fleet was at its peak of development [49], with a range of 0.2–0.56, corresponding to overexploitation. In the case of *D. oxyrinchus*, we only used the initial B/k prior (2004), set at 0.1–0.26, corresponding to overexploitation. This value was set lower than the 2000 prior for *R. clavata* due to the higher sensitivity assumed for *D. oxyrinchus*. This assumption is primarily based on its larger body size, which is a known proxy for greater vulnerability to fishing pressure and extinction risk in batoids [51,52], as well as its slower life-history traits exemplified by a higher length at maturity and lower fecundity [53–55]. These traits are consistent with its classification as a species with low intrinsic resilience [48] and very high fishing vulnerability [56]. Finally, this assumption is further supported by the severe declines of closely related species, such as the blue skate (*Dipturus batis*) and the flapper skate (*Dipturus intermedius*), which were once common in north-western Europe but are currently considered critically endangered by the IUCN [57].

The LBB method analyses length frequency data and was originally designed for use with commercial catch data. However, since the selectivity for skates is similar between commercial gear and the MEDITS survey gear, we used data from the MEDITS surveys instead, as it reliably represents the exploited size distribution of the stock. This substitution is valid because the selectivity for skates is similar between gears. Specifically, while the commercial fleet uses a squared 42 mm mesh codend and MEDITS surveys use a diamond 20 mm mesh, literature shows that the 42 mm mesh demonstrates no selectivity for batoids, even for the smallest individuals [58]. This approach is particularly appropriate for *R. polystigma* in the Balearic Islands, for which catch data is unreliable due to frequent misidentification, high discard rates linked to its smaller size, and its grouping with other species under the same commercial category. Length-frequency data for *R. polystigma* were collected annually between 2002 and 2024 during the MEDITS surveys, which are conducted in the same fishing grounds and with the same selectivity regarding skates as the commercial fleet. These length frequencies were standardised to individuals per km^2^. Then, the weighted average of density by size class was calculated considering the surface of each depth strata. To ensure representative size distributions of the population with sufficient individuals, data were aggregated into 3 cm size ranges and grouped into 6-year periods, except for the latest period (2020–2024), which comprises 5 years (S1 Fig). LBB does not require additional input data, making it suitable for data-poor stocks [35]. The model estimates the asymptotic length (L_inf_), the length at first capture where 50% of the individuals are retained by the gear (L_c_), natural mortality rate relative to somatic growth rate (M/K), ﬁshing mortality rate relative to somatic growth rate (F/K), mean relative fishing mortality (F/M, which here can be considered as a proxy for F/F_MSY_) and current biomass relative to unﬁshed biomass (B/B_0_) [35]. The LBB routine also provides other estimates as the length where the biomass of an unexploited cohort would be maximum (L_opt_) and the length at first capture that maximises catch and biomass for a given fishing pressure (Lc_opt_) [59].

## Results

### Conservation status

We used a total set of 181 sequences of the mitochondrial COI gene (149 new sequences and 32 sequences previously obtained) from the Balearic Islands: 29 sequences of 591 base pairs (bp) for *D. oxyrinchus*, 27 sequences of 591 bp for *L. naevus*, 31 sequences of 591 bp for *R. brachyura*, 29 sequences of 582 bp for *R. miraletus*, 37 sequences of 590 bp for *R. polystigma* and 28 sequences of 591 bp for *R. radula* (Table 1).

In the case of *D. oxyrinchus* and *R. polystigma* only 1 haplotype was found in the study area; therefore, neither haplotype nor nucleotide diversity could be estimated for them (Table 2). Three haplotypes were found for *L. naevus, R. miraletus* and *R. radula* with haplotype diversities (h ± SD) ranging between 0.14 ± 0.08 and 0.21 ± 0.1 and nucleotide diversities (π ± SD) ranging from 0.0004 ± 0.0005 to 0.0008 ± 0.0008 (Table 2). *Raja brachyura* presented the highest h and π of all studied species, with 0.68 ± 0.07 and 0.0014 ± 0.0012, respectively (Table 2).

**Table 2:** Genetic diversity indices for the Cytochrome C Oxidase subunit I from different Rajidae species from the Balearic Sea (western Mediterranean).

Species	N	Nh	Nh_a_	h ± SD	π ± SD
*Dipturus oxyrinchus*	29	1	–	0	0
*Leucoraja naevus*	27	3	–	0.2108 ± 0.1005	0.0008 ± 0.0008
*Raja brachyura*	31	6	4	0.6753 ± 0.0681	0.0014 ± 0.0012
*Raja miraletus*	29	3	2	0.1355 ± 0.0845	0.0004 ± 0.0005
*Raja polystigma*	37	1	–	0	0
*Raja radula*	28	3	3	0.1402 ± 0.0871	0.0005 ± 0.0006
Raja clavata*	31	2	1	0.125 ± 0.077	0.0002 ± 0.0004

N: number of samples, Nh: number of haplotypes, Nh_a_: number of haplotypes exclusive to the area, h: haplotype diversity; and π: nucleotide diversity. The two diversity indicators are shown with their corresponding standard deviations (SD). *: Indices obtained by Ferragut-Perello et al. [33].

The dataset of genetic diversity indices used to calculate a comparative framework included 42 populations of 18 Rajidae species from the Mediterranean Sea and the Atlantic Ocean (Table 3) [60–68]. The resulting median value of π of the comparative framework was 0.001 (95% CI: 0.0005–0.0019). The π median and its 95% CI were slightly lower for the Mediterranean populations of the comparative framework (0.001 (95% CI: 0.0003–0.0023)) than for the Atlantic populations (0.0014 (95% CI: 0.0003–0.0027)), although they mostly overlapped.

**Table 3:** Comparative framework dataset of available population genetics information of different Rajidae species from the Mediterranean Sea and the Atlantic Ocean.

Species	Area	Subarea	N	Nh	h ± SD	π ± SD	Reference
*Amblyraja hyperborea*	Atlantic	Northeast Atlantic	10	2	0.2	0.0003	[60]
*Amblyraja hyperborea*	Atlantic	Canada	30	7	0.582	0.0014	[61]
*Amblyraja jenseni*	Atlantic	Canada	20	5	0.663	0.0017	[61]
*Amblyraja radiata*	Atlantic	Northeast Atlantic	10	8	0.956	0.005	[60]
*Amblyraja radiata*	Atlantic	Canada	38	20	0.939	0.0071	[61]
*Bathyraja spinicauda*	Atlantic	Canada	29	4	0.253	0.001	[61]
*Dipturus nidarosiensis*	Atlantic	Northeast Atlantic	10	2	0.556	0.0009	[60]
*Dipturus nidarosiensis*	Mediterranean	Sardinia	49	3	0.119 ± 0.062	0.0002 ± 0.0003	[62]*
*Dipturus oxyrinchus*	Mediterranean	Malta	10	2	0.2 ± 0.154	0.0003 ± 0.0005	[63]
*Dipturus oxyrinchus*	Mediterranean	Sardinia	175	11	0.253 ± 0.044	0.0005 ± 0.0001	[64]
*Dipturus oxyrinchus*	Atlantic	Northeast Atlantic	10	1	0	0	[60]
*Dipturus oxyrinchus*	Mediterranean	Ionian Sea	14	1	0	0	[65]*
*Leucoraja erinacea*	Atlantic	Canada	67	30	0.88	0.0049	[61]
*Leucoraja ocellata*	Atlantic	Canada	35	16	0.93	0.0033	[61]
*Malacoraja senta*	Atlantic	Canada	44	7	0.686	0.0017	[61]
*Raja asterias*	Mediterranean	North Africa	30	6	0.559 ± 0.088	0.0186 ± 0.0163	[66]
*Raja asterias*	Mediterranean	Sardinia + Tyrrhenian Sea	138	7	0.1287 ± 0.0663	0.0039 ± 0.0064	[66]**
*Raja asterias*	Mediterranean	Sicilian Strait + West Ionian	19	6	0.58 ± 0.1685	0.046	[66]**
*Raja asterias*	Mediterranean	Adriatic Sea	78	8	0.482 ± 0.1265	0.015 ± 0.0147	[66]**
*Raja brachyura*	Mediterranean	Sardinia	11	2	0.3273 ± 0.1533	0.0005 ± 0.0006	[65]*
*Raja clavata*	Mediterranean	Malta	25	2	0.08 ± 0.07	0.0001 ± 0.0003	[63]
*Raja clavata*	Atlantic	Northeast Atlantic	10	1	0	0	[60]
*Raja clavata*	Mediterranean	Adriatic Sea	40	2	0.05 ± 0.0469	0.0001 ± 0.0002	[65]*
*Raja miraletus*	Atlantic	South Africa	41	9	0.691 ± 0.063	0.0019 ± 0.0035	[67]
*Raja miraletus*	Atlantic	Angola	27	10	0.858 ± 0.041	0.0254 ± 0.0196	[67]
*Raja miraletus*	Atlantic	Portugal	10	1	0	0	[67]
*Raja miraletus*	Mediterranean	Sardinia	11	1	0	0	[67]
*Raja miraletus*	Mediterranean	North Tyrrhenian	28	2	0.701 ± 0.065	0.0001 ± 0.0005	[67]
*Raja miraletus*	Mediterranean	Algerian Coasts	17	4	0.419 ± 0.141	0.0014 ± 0.0022	[67]
*Raja miraletus*	Mediterranean	Sicilian Strait	45	6	0.711 ± 0.039	0.0029 ± 0.0026	[67]
*Raja miraletus*	Mediterranean	Adriatic Sea	87	7	0.486 ± 0.051	0.001 ± 0.0023	[67]
*Raja miraletus*	Mediterranean	Israel	14	2	0.527 ± 0.064	0.001 ± 0.0006	[67]
*Raja miraletus*	Mediterranean	Adriatic Sea	20	3	0.4684 ± 0.1045	0.0009 ± 0.0009	[65]
*Raja montagui*	Atlantic	Western Irish Sea	30	5	0.193 ± 0.095	0.0003 ± 0.0002	[68]
*Raja polystigma*	Mediterranean	Sicilian Strait	10	4	0.778 ± 0.091	0.002 ± 0.0003	[68]
*Raja polystigma*	Mediterranean	Tyrrhenian Sea	32	6	0.701 ± 0.0815	0.0023 ± 0.0003	[68]
*Raja polystigma*	Mediterranean	Sardinia	37	9	0.8255 ± 0.0515	0.0025 ± 0.0003	[68]
*Raja polystigma*	Mediterranean	Algerian Coasts	13	4	0.654 ± 0.106	0.0019 ± 0.0003	[68]
*Raja radula*	Mediterranean	Malta	22	3	0.437 ± 0.105	0.0008 ± 0.0008	[63]
*Rajella fyllae*	Atlantic	Northeast Atlantic	10	4	0.778	0.0027	[60]
*Rajella fyllae*	Atlantic	Canada	19	1	0	0	[61]
*Rajella lintea*	Atlantic	Northeast Atlantic	10	1	0	0	[60]

N: number of samples, Nh: number of haplotypes; h ± SD: haplotype diversity ± standard deviation when available; π ± SD: nucleotide diversity ± standard deviation when available. *: indices calculated from available sequences. **: indices recalculated regarding COI population structure.

**Fig 2:**
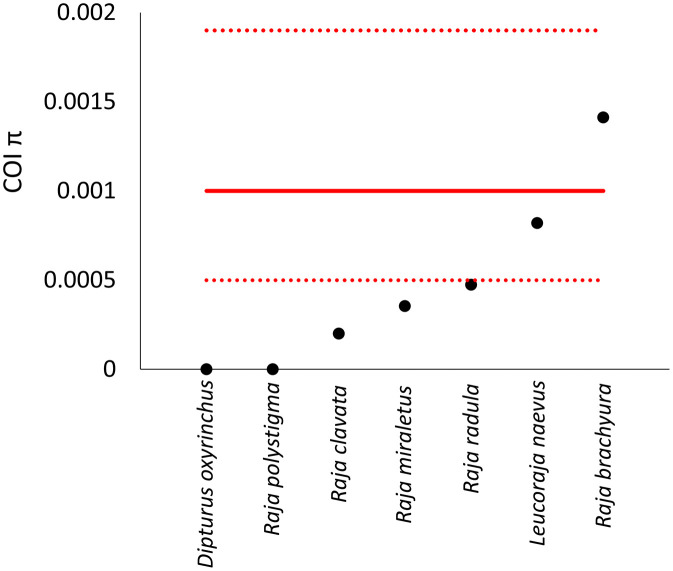
Levels of nucleotide diversity (π) in Cytochrome C Oxidase subunit I (black dots) of different Rajidae species from the Balearic Sea (western Mediterranean). The red solid line shows the median nucleotide diversity value obtained by bootstrapping a dataset containing π values from the Atlantic and Mediterranean Rajidae populations (comparative framework). Dotted lines show the corresponding 95% confidence intervals.

The π values of four out of the seven studied species in the Balearic Islands (*D. oxyrinchus, R. polystigma, R. clavata* and *R. miraletus*) were below the lower CI boundary of the median of the comparative framework, whereas two of them remained within the 95% CI range (*L. naevus* and *R. brachyura*) (Fig 2). The π value of *R. radula* was positioned on the lower boundary of this 95% CI (Fig 2).

For *D. oxyrinchus* and *R. polystigma*, the single haplotype identified in the Balearic Islands for each species was shared with all other analysed populations. *Dipturus oxyrinchus* shared its haplotype with populations from the Ionian Sea, Atlantic Ocean, Malta, and Sardinia, while *R. polystigma* shared its haplotype with those from Algeria, Sardinia, the Strait of Sicily, and the Tyrrhenian Sea. In *R. clavata*, one of the two haplotypes detected in the Balearic Islands was the dominant haplotype, shared across all analysed populations (Adriatic Sea, Malta, and Northeast Atlantic), whereas the second haplotype was exclusive to the study area. For *R. miraletus*, two out of the three haplotypes found in the Balearic Islands were unique, while the third was shared with five of the ten studied populations (Adriatic Sea, Sicily, Portugal, North Tyrrhenian Sea, and Algeria). In the case of *R. brachyura*, four out of the six identified haplotypes were exclusive to the area, whereas the remaining two were shared with the population from Sardinia. Finally, all three haplotypes identified for *R. radula* in the Balearic Islands were exclusive to this area. No haplotype comparisons could be made for *L. naevus* due to the lack of sequence data that met the inclusion criteria for the dataset used to calculate the comparative framework.

### Exploitation status

**Fig 3:**
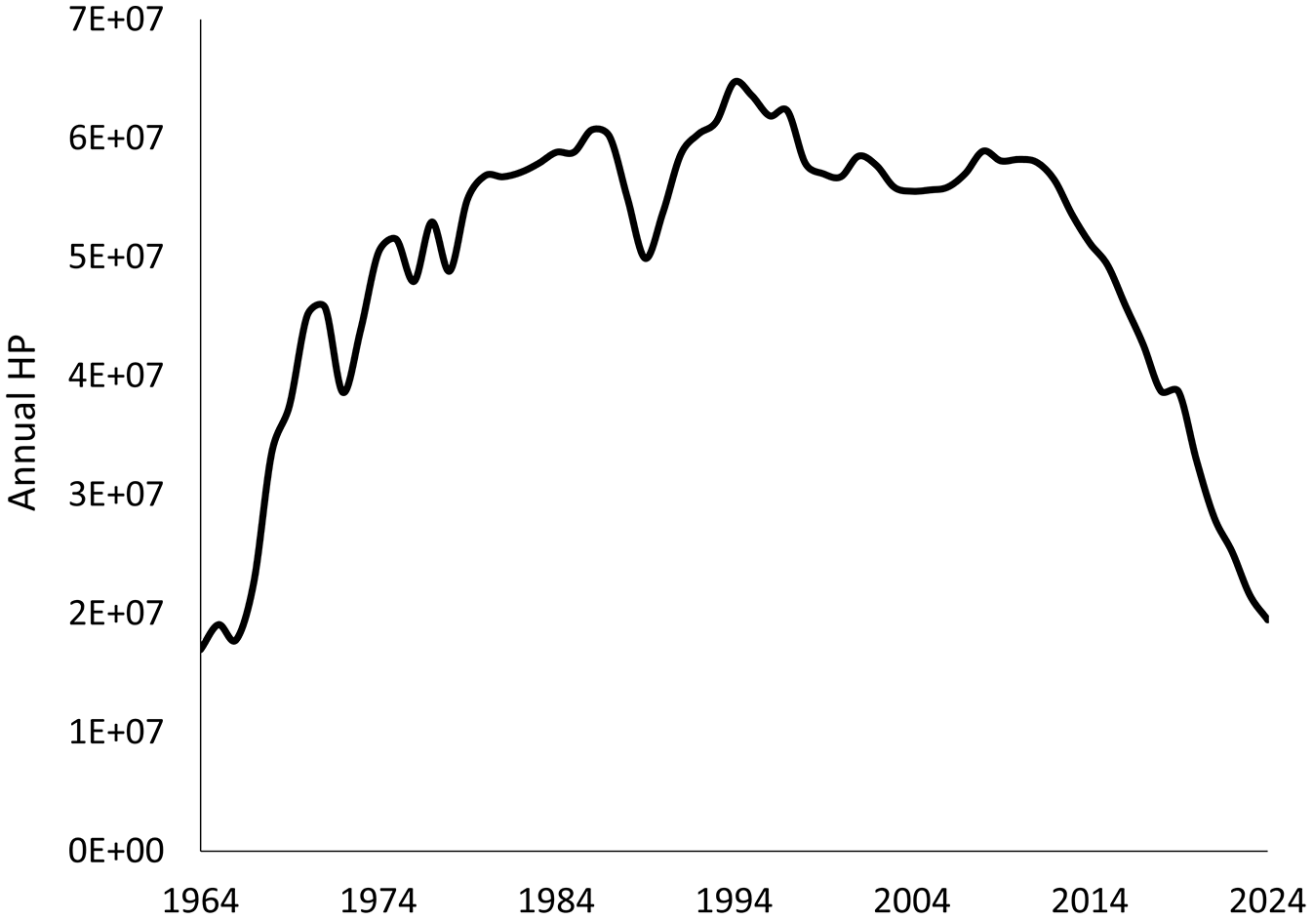
Annual engine horse power (HP) of the bottom trawling fleet from the Balearic Islands (western Mediterranean) between 1964 and 2024.

The fishing effort of the bottom trawl fleet from the Balearic Islands has shown important variations during 1964–2024 (Fig 3), with a very sharp increase from the start of the time series until the mid-1990s and, after a quite stable period until 2012, it decreased also very abruptly up to the present.

**Fig 4:**
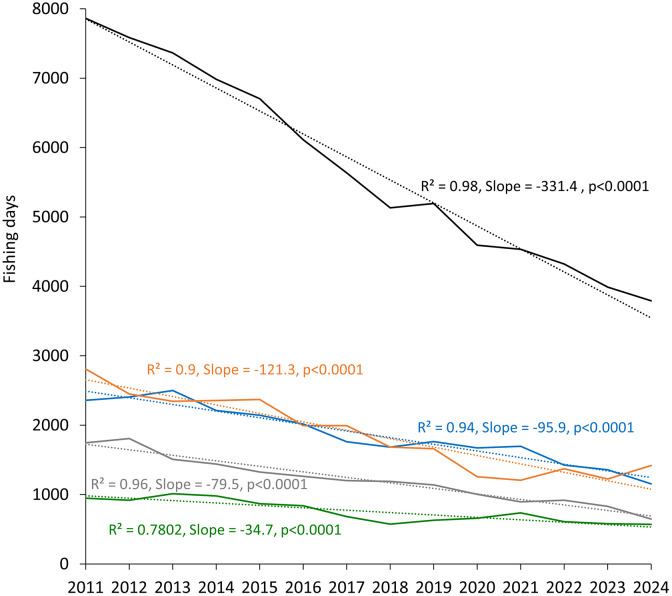
Time series of fishing effort (fishing days) of the bottom trawl fleet calculated from VMS data by bathymetric strata. Dotted lines correspond to significant linear regressions, coloured as follows: black, 50–800 m; blue, 50–100 m; green, 100–200 m; grey, 200–500 m; orange, 500–800 m. R^2^: coefficient of determination; p: significance level.

The evolution of the fishing days in total and by depth strata showed significant linear decreasing trends (Fig 4). The most important reduction has occurred in the middle slope (500–800 m depth), where the fleet targets blue and red shrimp. From 2020 onward, a continued decline in fishing days was observed in the shelf (from 1674 to 1154 days), shelf-break (from 658 to 571), and upper slope (from 1003 to 646). In contrast, the middle slope showed a slight recent increase, from 1258 days in 2020 to 1419 in 2024.

**Fig 5:**
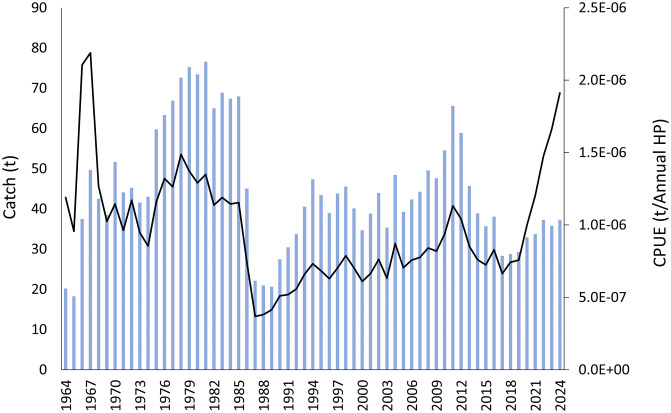
Catch (blue bars) and CPUE (black line) of *Raja clavata* from the Balearic Islands (western Mediterranean) between 1964 and 2024.

The CPUE of *Raja clavata* was high from 1964 to mid-1980s, with a marked peak in 1967–1968, but decreased abruptly up to 1987–1989, when the lowest values of the time series were reached (Fig 5). From this minimum, both catches and CPUEs began a gradual upward trend until 2011. After a short decline until 2017–2018, the catches remained rather stable, but CPUEs increased very sharply up to the present, reaching values comparable to the maximum ones observed at the beginning of the time series.

**Fig 6:**
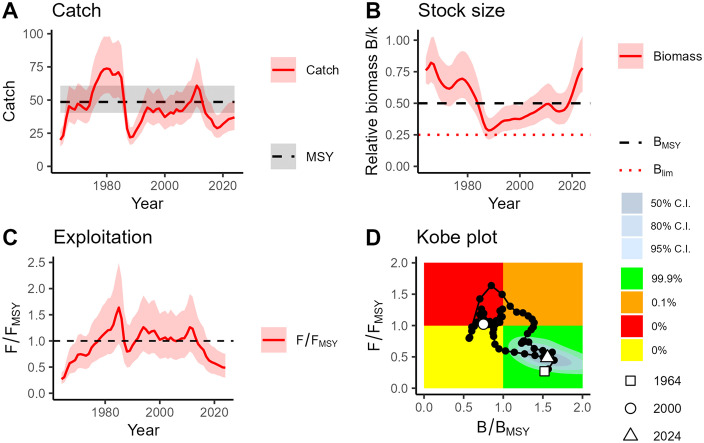
Stock dynamics of *Raja clavata* from the Balearic Islands (western Mediterranean) estimated by the BSM model, showing the catch (A), the relative biomass (B), the relative exploitation rate (C) and the Kobe plot (D). Colour code of the Kobe plot: red (top-left quadrant; overfishing and overfished stock); orange (top-right quadrant; healthy stock size with overfishing); yellow (bottom-left quadrant; reduced fishing pressure on stock recovering from low biomass level); green (bottom-right quadrant; sustainably exploited stock). Percentages represent the posterior probability of stock status in the final year, estimated as the proportion of Bayesian model simulations falling within each Kobe plot quadrant. C.I.: confidence intervals.

According to the BSM results, the catches of *Raja clavata* have only been above the values obtained fishing at the MSY on two occasions (1978–1986 and 2009–2013) during the available time series (Fig 6A). The stock relative biomass (B/k) showed a decreasing trend from the start of the time series up to the late 1980s, when the lowest historical value was attained; since then, it showed a gradual increasing trend, which was markedly accelerated in the recent years, that drove relative biomass above the B_MSY_ (Fig 6B). The relative exploitation pattern (F/F_MSY_) increased sharply since 1964, driving the stock from the underexploitation state to the highest values of overexploitation of the time series in 1985; this maximum was followed by a sharp decrease in F/F_MSY_ values, which remained close to 1 up to 2011, but decreased again to the underexploitation state from 2018 up to the present (Fig 6C). According to the Kobe plot (Fig 6D), *R. clavata* was exploited sustainably (green area) for nearly 15 years after the start of the time series, but then was overexploited for approximately 35 years (orange and red areas); in the last decade, the stock returned to the sustainable state as a result of both the fishing pressure decrease and biomass increase. The estimated F/F_MSY_ value in the final year was 0.49, with a 95% confidence interval of 0.30–0.75. The estimated B/B_MSY_ value in the final year was 1.56 (95% CI: 1.18–2.06). The r–K parameter space and the prior/posterior distributions of the BSM for *R. clavata* are presented in S2 Fig and S3 Fig, respectively.

**Fig 7:**
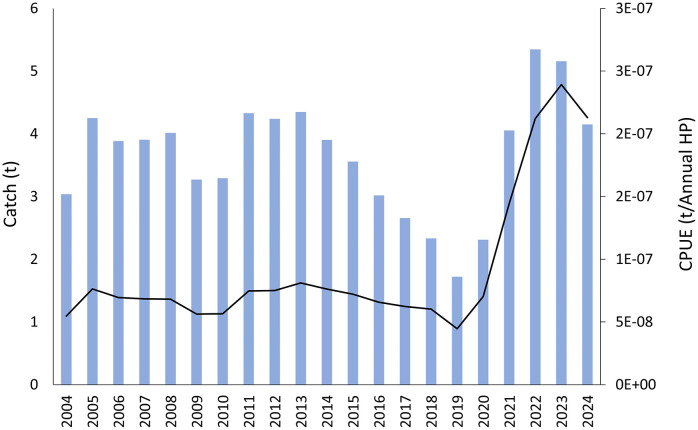
Catch (blue bars) and CPUE (black line) for *Dipturus oxyrinchus* from the Balearic Islands (western Mediterranean) between 1964 and 2024.

The CPUE of *D. oxyrinchus* remained stable between 2004 and 2019, but then increased sharply up to a historical peak in 2023 (Fig 7). Catches were also rather stable from 2004 to 2013, but decreased gradually up to the historical minimum in 2019; like CPUEs, catches increased afterwards reaching the highest values in 2022–2023 (Fig 7).

**Fig 8:**
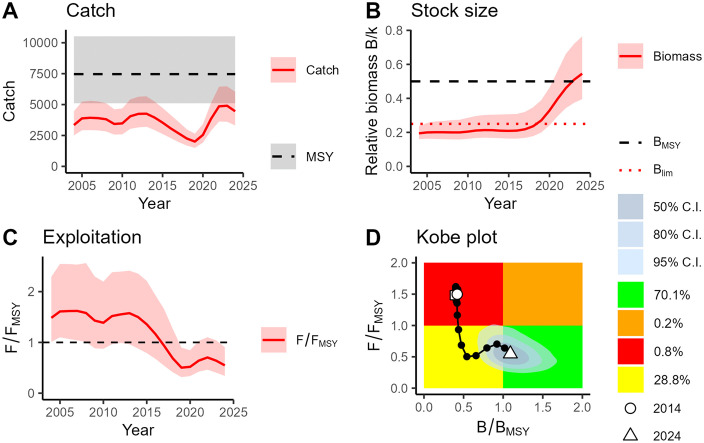
Stock dynamics of *Dipturus oxyrinchus* from the Balearic Islands (western Mediterranean) estimated by the BSM model, showing the catch (A), the relative biomass (B), the relative exploitation rate (C) and the Kobe plot (D, see colour code in Fig 5). Percentages represent the posterior probability of stock status in the final year, estimated as the proportion of Bayesian model simulations falling within each Kobe plot quadrant. C.I.: confidence intervals.

The BSM results obtained for *D. oxyrinchus* revealed that catches have remained well below the MSY levels for the entire time series, though they showed an important increase from 2019 up to the present (Fig 8A). The relative biomass (B/k) displayed a very slight increasing trend between 2004 and 2016, followed afterwards by such a sharp increase that brought B/k values exceed the B_MSY_ reference point in 2023 (Fig 8B). The relative exploitation pattern (F/F_MSY_) revealed that the stock was overexploited until 2016 but then it improved and has remained at the underexploitation state (F/F_MSY_ = 0.55) up to 2024 (Fig 8C). This improvement was also shown by the Kobe plot (Fig 8D), which revealed the overexploitation state until 2016 (red area), followed by a sharp drop in fishing pressure between 2014 and 2020, that brought the stock to the current sustainable state. The estimated F/F_MSY_ value in the final year was 0.55, with a 95% confidence interval of 0.34–0.91. The estimated B/B_MSY_ value in the final year was 1.09 (95% CI: 0.79–1.53). The r–K parameter space and the prior/posterior distributions of the BSM for *D. oxyrinchus* are presented in S4 Fig and S5 Fig, respectively.

**Fig 9:**
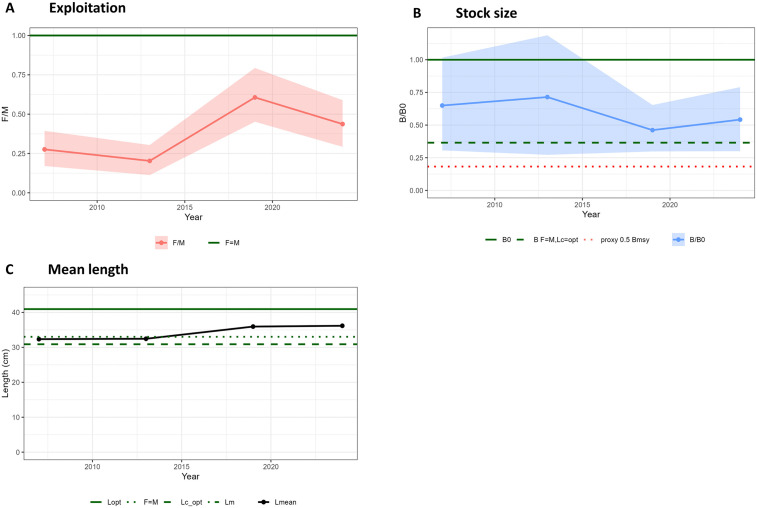
Stock dynamics of *Raja polystigma* from the Balearic Islands (western Mediterranean) estimated by the LBB model, showing A) the relative exploitation rate (F/M) with 95% conﬁdence limits, with indication of the reference level where F = M (horizontal line); B) the relative biomass (B/B0) with 95% conﬁdence limits, with indication of a proxy for Bmsy (dashed line) and a proxy for 0.5 Bmsy (dotted line); and C) the catch mean length (Lmean) relative to the length where the biomass of an unexploited cohort would be maximum (Lopt), and Lc_opt, which determines the Lc (the length at first capture where 50% of the individuals are retained by the gear) value that would result in L_opt_ becoming the mean length in the catch.

The LBB results for *R. polystigma* revealed fluctuations of the F/M ratio throughout the entire time series but with values well below 1, which is considered a proxy of F_MSY_, and the curent F/M being 0.44 (Fig 9A). The length at first capture (L_c_) resulted in 24 cm. The relative biomass to the unfished biomass (B/B_0_) fluctuated above the biomass predicted when F = M and Lc = Lcₒₚₜ (used as a proxy for B_MSY_), with the most recent value of B/B_0_ (0.54) remaining above this B_MSY_ (0.37) (Fig 9B). The mean length of the catch (L_mean_) followed an increasing trend and remained above the optimum length at first capture (Lc_opt_, 31 cm) since the start of the time series. In the most recent period, L_mean_ (36 cm) was above Lcₒₚₜ and close to the Lₒₚₜ (41 cm) (Fig 9C).

## Discussion

The conservation and exploitation status of Rajidae species in the Balearic Islands, based on genetic diversity analysis and stock assessment models, reveals critical insights into the resilience and management needs of these vulnerable elasmobranch populations. Our results show interspecific variability in genetic diversity and different exploitation patterns.

The nucleotide diversity values estimated here showed that all species, with the exception of *L. naevus* and *R. brachyura*, fell below the confidence intervals of the median nucleotide diversity based on the Mediterranean–Atlantic Rajidae comparative framework. This indicates that populations of said species in the Balearic Islands, in general, seem to be in an inferior conservation status compared to the collective Mediterranean and Atlantic populations analysed. There was not a clear relationship between local diversity values and IUCN categories: while Endangered species (i.e., *R. radula*) were located at the lower end of the confidence intervals, the other species, classified as Near Threatened (*D. oxyrinchus, R. clavata* and *L. naevus*) and Least Concern (*R. polystigma* and *R. miraletus*) were distributed indiscriminately above and below the confidence intervals (Fig 2). However, the number of species analysed in this study is low to conclude that COI-π match the expectations from IUCN list.

As expected, the nucleotide diversity values estimated here are much lower than those from teleost fish species exploited in the Balearic Islands (e.g., *Lophius budegassa, Merluccius merluccius, Mullus barbatus* and *Mullus surmuletus*; [37]). It is known that, as a result of fundamental differences in life-history traits, teleosts exhibit consistently higher nucleotide diversity values compared to elasmobranchs [69,70]. In general, teleosts have life-history traits that contribute to greater levels of genetic variation such as shorter generation times, higher fecundity, and larger effective population sizes [70]. By contrast, sharks and batoids are characterized by slow growth, late maturity, and low reproductive output, resulting in reduced effective population sizes and lower nucleotide diversity [71]. Comparative studies have shown that elasmobranchs not only possess slower evolutionary rates of mitochondrial genes [72] but also exhibit lower levels of genomic polymorphism compared to teleosts [73]. These molecular traits could cause that, although some batoid populations remain stable or are recovering, they currently have a low genetic diversity due to past events that delay their genetic recovery [74,75]. The low genetic diversity could also be linked to behavioural effects such as high site fidelity and residency [76].

The skates *D. oxyrinchus* and *R. polystigma* did not display nucleotide nor haplotype diversities. Similar results were recorded for the first species in the Ionian Sea [65] and the northeast Atlantic [60]. Although the species showed higher genetic diversity in Malta and Sardinia [63,64], their values were relatively low, especially compared with other vertebrates [72]. On the other hand, the populations of *R. polystigma* considered in the comparative framework displayed higher π values, close to 0.002, and between 4 and 9 haplotypes per area [68], highlighting the need to locally assess conservation status for “low-migrant” species, particularly from isolated areas such as the Balearic Islands.

Although *R. clavata* from our study area showed low genetic diversity values within the framework of the Rajidae comparison, its value was higher than in other Mediterranean regions such as Malta [63] and the Adriatic Sea (calculated from [65]). A consistent pattern was observed across all three Mediterranean areas: each one exhibiting a single haplotype unique to the region, as well as one shared haplotype. This suggests that while the Mediterranean population may be broadly connected, local adaptation could lead to non-uniform resilience to stressors across its range.

The low or non-existent nucleotide diversity observed in these three species supports the hypothesis that overfished species exhibit reduced genetic variability [36,77]. Although currently *D. oxyrinchus* and *R. clavata* show a sustainable exploitation status in the Balearic Islands, both in terms of fishing pressure and biomass, their genetic diversity might still not be recovered from the recent overexploitation events found in our analysis. This sustainable status is clearly indicated by the stock assessment models, even when considering estimation uncertainty. For *R. clavata*, the 95% confidence intervals for both biomass and fishing mortality place its stock status within the healthiest quadrant of the Kobe plot. For *D. oxyrinchus*, the intervals confirm a sustainable exploitation situation, with the range of plausible values indicating that biomass is either close to or has already recovered above the B_MSY_ reference point. Similarly, the situation of *R. polystigma* could also be linked to a period of high exploitation period in the past, although with our relatively shorter 20-year time series we were unable to detect it. Our results show that the length at first catch is lower than the optimal length at first catch, meaning smaller individuals are being caught. This prevents cohorts from reaching their maximum biomass and is consistent with these species lack of selectivity to trawl gear. In fact, whereas Ferragut-Perello et al. [78] detected a stable trend for conservation status indicators of this species in the Balearic Islands during the last two decades, they also reported a spatial distribution pattern with lower abundance in areas with higher bottom trawl fishing effort, hence, highlighting the vulnerability of this species to fishing activities. Therefore, *R. polystigma* may have undergone a history of fishing exploitation similar to *R. clavata*, a species with which it overlaps in bathymetric distribution [13,14].

The low genetic diversity values could also be related with the evolutionary history of each species, as revealed by historical demographic analyses of *R. clavata* and *D. oxyrinchus* Mediterranean populations. Chevolot et al. [79] found significant genetic differentiation between Atlantic and Mediterranean populations of *R. clavata*, with mitochondrial DNA data suggesting a historical bottleneck as a result of a post-glacial recolonization of the Mediterranean from a limited number of founders. Similarly, *D.* *oxyrinchus* showed strong phylogeographic structure, with no shared haplotypes between Atlantic and Mediterranean populations, supporting long-term isolation and limited gene flow since their divergence approximately 20,000 years ago [80]. Although direct evidence of historical bottlenecks in *R. polystigma* is currently lacking, recent studies have revealed significant population structure and genetic differentiation across the Mediterranean [68]. The phylogeographic patterns observed in these three species are consistent with low effective population sizes and limited dispersal capacity, which, combined with recent anthropogenic pressures such as overfishing, may further erode their genetic diversity and adaptive potential. In this sense, a long period of overexploitation and loss of genetic diversity may have left the extant populations with many missing alleles [81]. Nonetheless, the use of an extended dataset of molecular markers, such as genomic SNPs, is needed to further investigate this hypothesis by demographic analyses.

On the other hand, in species having relatively large population sizes and apparently good conservation status, the low genetic variation may be related to a recent speciation process [82]. In recent species the gene pool is limited, and genetic drift can rapidly reduce variation [83]. Additionally, if the species emerged following a population bottleneck or a founder effect, the loss of allelic richness and heterozygosity is expected [84]. The short evolutionary timespan since divergence also limits the accumulation of new mutations, further contributing to reduced genetic variability [83,84]. This low diversity can have important implications for the species’ adaptability and long-term viability, especially under environmental change or anthropogenic pressures [85]. This could be the case of *R. polystigma*, a species that originated recently during the Pleistocene in the Mediterranean [15,86]. Our results on its current exploitation status in the Balearic Islands aligns with its Least Concern consideration by the IUCN and with the stable and even increasing trends detected in various indicators of conservation status reported in the study area [19,78] and other Mediterranean areas such as Sardinia [87]. Taken together, these factors suggest the population of *R. polystigma* in the study area is in good conservation status despite the lack of nucleotide diversity. However, this lack of diversity should be monitored closely, as it may limit the population’s ability to adapt to future environmental changes or other stressors.

The species inhabiting the shallow continental shelf (*R. brachyura, R. miraletus,* and *R. radula*) exhibited differences in genetic diversity in the Balearic Islands. The skate *R. brachyura* showed the highest nucleotide diversity (π), exceeding the lower confidence interval (LCI) of the Rajidae comparative framework median and being an order of magnitude greater than values reported from Sardinia [65]. This high genetic diversity likely relates to its coastal distribution, being restricted to depths above 60 m in the study area [14] and thus showing the most limited exposure to trawling among the analysed species. By contrast*, R. miraletus* showed lower π values (below the comparative framework’s LCI), though comparable to Adriatic and North Tyrrhenian populations, but higher than Sardinia and lower than the remaining analysed populations of this species [65,67]. The π of *R. radula* fell just at the median LCI, showing values similar to those reported in Malta [63]. In general, these last three species displayed a high proportion of haplotypes exclusive to the area, with *R. radula* presenting entirely unique haplotypes. This pattern suggests that these species have restrictive genetic flow with other Mediterranean areas, which could be due to an isolation by bathymetric barriers, as they are restricted to waters shallower than 150 m depth. The Balearic Islands are surrounded by steep continental slopes and deep marine basins [88] which could limit the dispersal of these species. Although an expanded sampling effort covering a wider geographic range and additional molecular markers are needed to confirm this, the possibility of isolated populations calls for a precautionary approach to the management of these species in the Balearic Islands and other insular areas with similar bathymetric barriers that could limit the exchange of individuals.

The skate *L. naevus* presented the second highest value of nucleotide diversity of the species analysed in the study area, exceeding the median LCI. While genetic comparisons with other Mediterranean populations remain unattainable due to the paucity of COI data elsewhere, the species frequency of occurrence was higher than in other Mediterranean areas [11], further supporting relatively better exploitation status. This agrees with the species high genetic diversity and, as in the previous skates, is also likely related to its bathymetric distribution. In the study area, the species optimum depth is around 164 m [14], inhabiting almost exclusively the deep shelf grounds where trawl fishing pressure is minimal [31].

In fact, apart from *L. naveus,* also other species, such as *R. clavata* [14] and the juvenile population of *R. polystigma* [78], have their optimum depths on the deep shelf grounds. Despite historical overexploitation and inherently vulnerable life-history traits, the stabilization, and in some cases recovery, of these populations may be attributed to the low overlap between their bathymetric distribution and the depth strata more intensively exploited by the bottom trawl fleet, which are the shallow shelf from 50 to 100 m and the middle slope from 500 to 800 m [31]. This would be the case for *R. clavata*, whose population has recovered after a long overexploitation period. In the Balearic Islands, this species has a broad bathymetric distribution, from 60 to 600 m depth with an optimum at 224 m [14]. In the case of *R. polystigma,* the adults inhabit the shelf grounds exploited by the trawling fleet, which would explain its lower genetic diversity compared to *R. clavata* and *L. naevus*. By contrast, the juveniles aggregate on the deep shelf bottoms [78], contributing to the current stability of the species population.

The analysis of commercial data results showed a remarkable recent increasing trend in CPUE for *R. clavata* and *D. oxyrinchus*, probably being linked to the considerable reduction in fishing effort observed in the study area in the last years. It is important to contextualize this reduction, as the fishing effort in the Balearic Islands has historically been much lower than in adjacent areas of the western Mediterranean [16]. Therefore, the recent effort reductions, while substantial in relative terms, are built on an already low baseline of exploitation. This interpretation is further supported by the analysis of effort distribution, which shows a significant reduction in fishing days across all bathymetric strata since 2011, a trend that has continued or stabilized following MAP implementation. Crucially, the only recent effort increase occurred in the middle slope, where the main target species is the red and blue shrimp and skate catches are negligible, confirming that the overall fleet activity has not redistributed into main skate habitats, providing support for the interpretation of the observed CPUE trends as signals of stock recovery. Additional support comes from fishery-independent data, as standardized biomass indices from MEDITS surveys show increasing trends for both species over the study period, consistent with the commercial CPUE patterns (S6 Fig). In the case of *R. clavata*, the current CPUE levels are similar to those recorded at the beginning of the fishery exploitation, when the stock was possibly still in a near-pristine state. The data strongly suggests that the stock is indeed recovering because such an increase in CPUE has occurred despite landings having remained stable, implying that the same biomass is being extracted with significantly less effort. In the case of *D. oxyrinchus*, the sharp increase in CPUE in recent years has been accompanied, apart from a reduced effort, by a substantial increase in landings, which points to a significant increase in the stock biomass.

Although the lack of data prevented determining the exploitation status of the species inhabiting the shallow shelf, previous results in the study area [19] suggest that *R. brachyura* and *R. miraletus* populations had good environmental status (GES). However, *R. radula* displayed contrasting results, as despite meeting most GES indicators, it showed a 15-year decline in the proportion of large individual’s indicator [19]. Paradoxically, the species remains abundant in the region, with occurrence frequencies in MEDITS surveys nearly twice those in the Aegean Sea, the second area with higher frequency of occurrence after Balearic Islands [11]. This discrepancy between local abundance and the Endangered IUCN classification highlights the importance of regional conservation strategies for elasmobranchs.

The significant population recoveries observed in *R. clavata* and *D. oxyrinchus,* as well as the apparent stability in the remaining species, suggest that factors beyond depth refuges are contributing to these trends. One possible explanation lies in their documented tolerance to trawling and air exposure, which may enhance their resilience. Notably, *R. clavata* has demonstrated short-term survival rates of approximately 60% even after one hour of trawling, indicating a considerable capacity to endure common fishing practices [89]. Moreover, physiological analysis showed that individuals surviving the capture process typically recover their homeostasis within 24 hours, as evidenced by normalised levels of key metabolites such as glucose, lactate and triglycerides in plasma and skin mucus [89]. This capacity to recover may play a crucial role in reducing fishing mortality of discarded individuals, thereby helping to explain the observed recovery or stability of these populations in the study area despite ongoing fishing pressure.

To conclude, the results presented here highlight the complex interplay between genetic diversity, bathymetric distribution, and fishing pressure in Rajidae populations off the Balearic Islands. Most populations showed genetic diversity below the Mediterranean-Atlantic comparative framework, though their capacity for recovery differs between species. Bathymetric distribution arises as a very important factor intervening in the populations’ resilience. While some species (e.g., *L. naevus, R. brachyura*) benefit from depth refuges that protect them from trawling impacts and enable them to preserve high genetic diversity, others (e.g., *D. oxyrinchus, R. clavata*) show signatures of genetic erosion (such as reduced nucleotide diversity) due to historical overexploitation despite recent recovery. Moreover, bathymetric isolation can create paradoxical scenarios, as for *R. radula*, whose bathymetric restriction to the shallow shelf has likely preserved local abundance through reduced trawl exposure, yet simultaneously promoted genetic differentiation through limited connectivity, resulting in exclusive haplotypes to the area. The discordance between its local abundance and its IUCN classification also emphasises the need for finer-scale assessments for proper elasmobranch conservation. Overall, our findings support the usefulness of genetic monitoring and the protection of critical depth ranges, acting as essential habitats, for managing batoid species. They also emphasise the importance of regional and species-specific conservation strategies for these species, given that their relatively low genetic diversity increases their vulnerability to environmental changes, limiting their adaptive potential, even in sustainably exploited populations, like those analysed in this work.

## Supporting information

S1 Fig Input length frequency distributions for every range of grouped years (blue dots). Annual LBB fits (red curve) with indication of median (bold green) and annual (dashed green) Linf and Lopt estimates.
(TIFF)

S2 Fig The graph shows as light grey dots the explored log r-k space in the BSM for *Raja clavata.* Black dots are viable r-k pairs found to be compatible with catch and CPUE data and the priors, with the most probable r-k pair indicted by the red cross (with CI). The dotted rectangle indicates the prior r-k range.
(TIFF)

S3 Fig Comparison of prior and posterior distributions for r, k, MSY and B/k in the BSM for *Raja clavata.* PPVR gives the ratio of posterior to prior variance.
(TIFF)

S4 Fig The graph shows as light grey dots the explored log r-k space in the BSM for *Dipturus oxyrinchus.* Black dots are viable r-k pairs found to be compatible with catch and CPUE data and the priors, with the most probable r-k pair indicted by the red cross (with CI). The dotted rectangle indicates the prior r-k range.
(TIFF)

S5 Fig Comparison of prior and posterior distributions for r, k, MSY and B/k in the BSM for *Dipturus oxyrinchus.* PPVR gives the ratio of posterior to prior variance.
(TIFF)

S6 Fig Standardized MEDITS survey biomass trends (2004–2024) for (a) *Raja clavata* and (b) *Dipturus oxyrinchus.* Linear regression lines (dotted) are shown with their coefficient of determination (R²) and p-values (*p*) for each species.
(TIF)
