# Supplementary figures and images for: Conservation and exploitation status of skate species (Batoidea: Rajidae) in the Balearic Islands, western Mediterranean
Source: PLoS One. 2026 Apr 29;21(4):e0347768. doi: 10.1371/journal.pone.0347768 (PMC13127908; doi:10.1371/journal.pone.0347768)

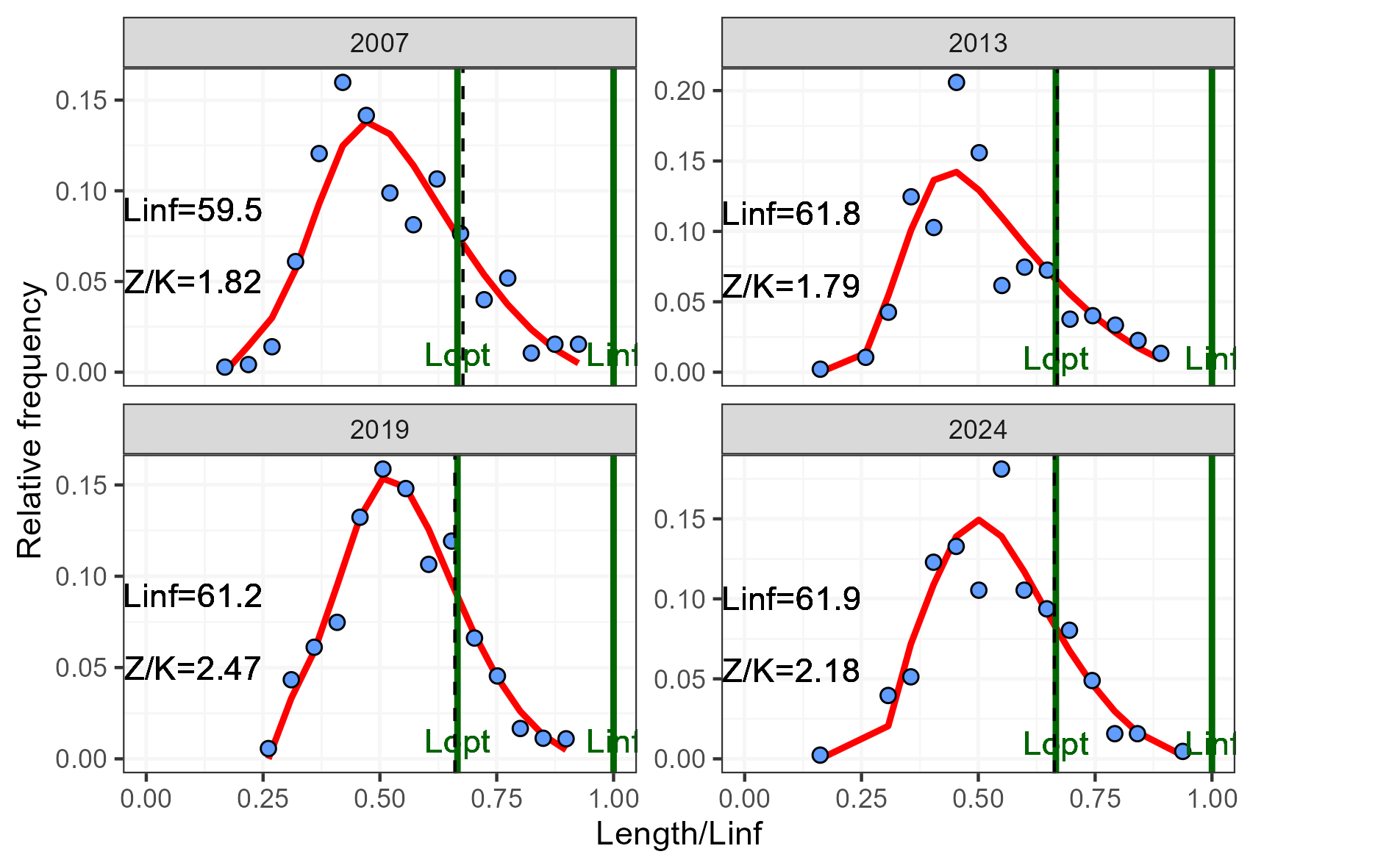

Supplement: S1 Fig — Annual LBB fits (red curve) with indication of median (bold green) and annual (dashed green) Linf and Lopt estimates. (TIFF) [file pone.0347768.s001.tiff]

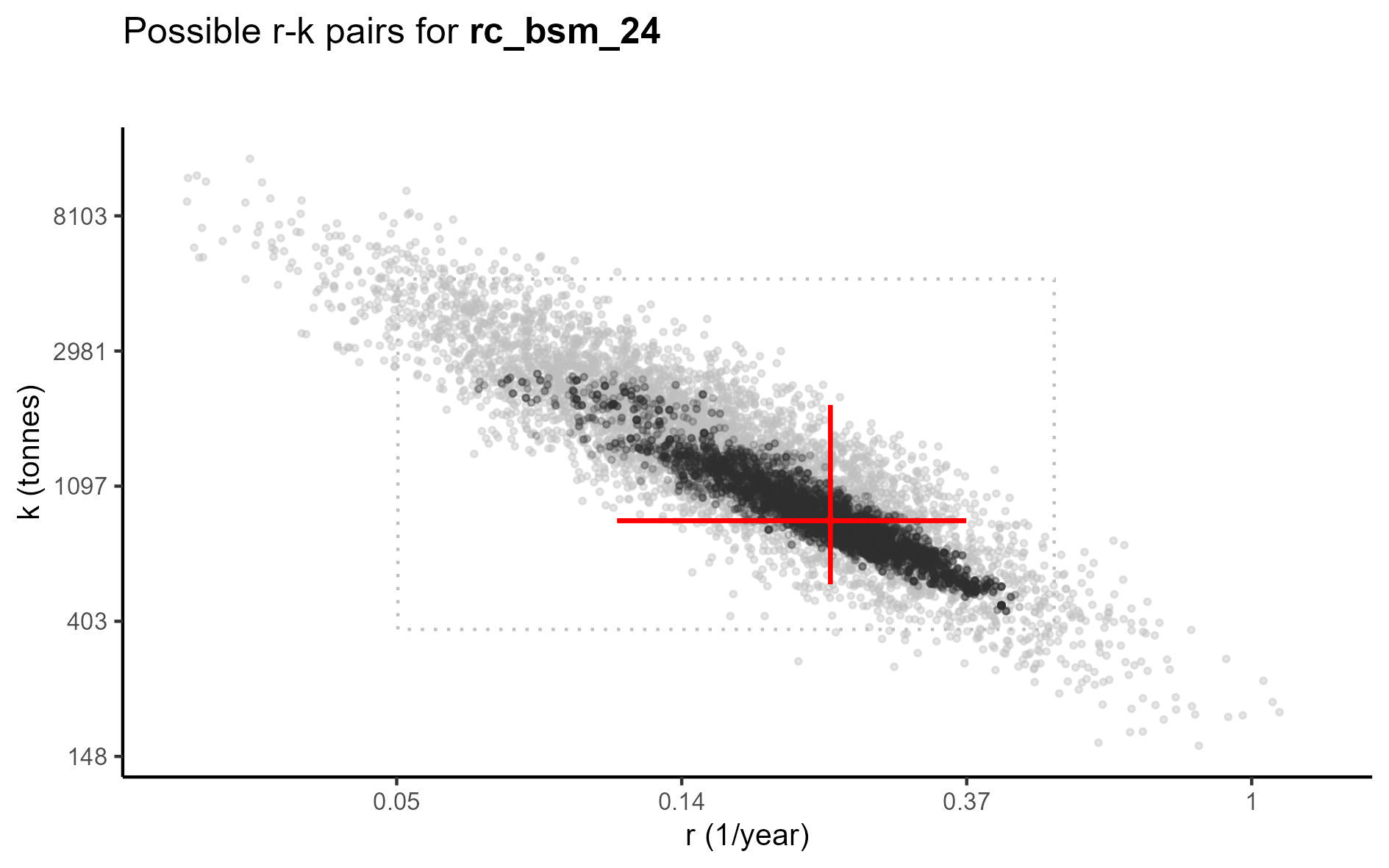

Supplement: S2 Fig — Black dots are viable r-k pairs found to be compatible with catch and CPUE data and the priors, with the most probable r-k pair indicted by the red cross (with CI). The dotted rectangle indicates the prior r-k range. (TIFF) [file pone.0347768.s002.tiff]

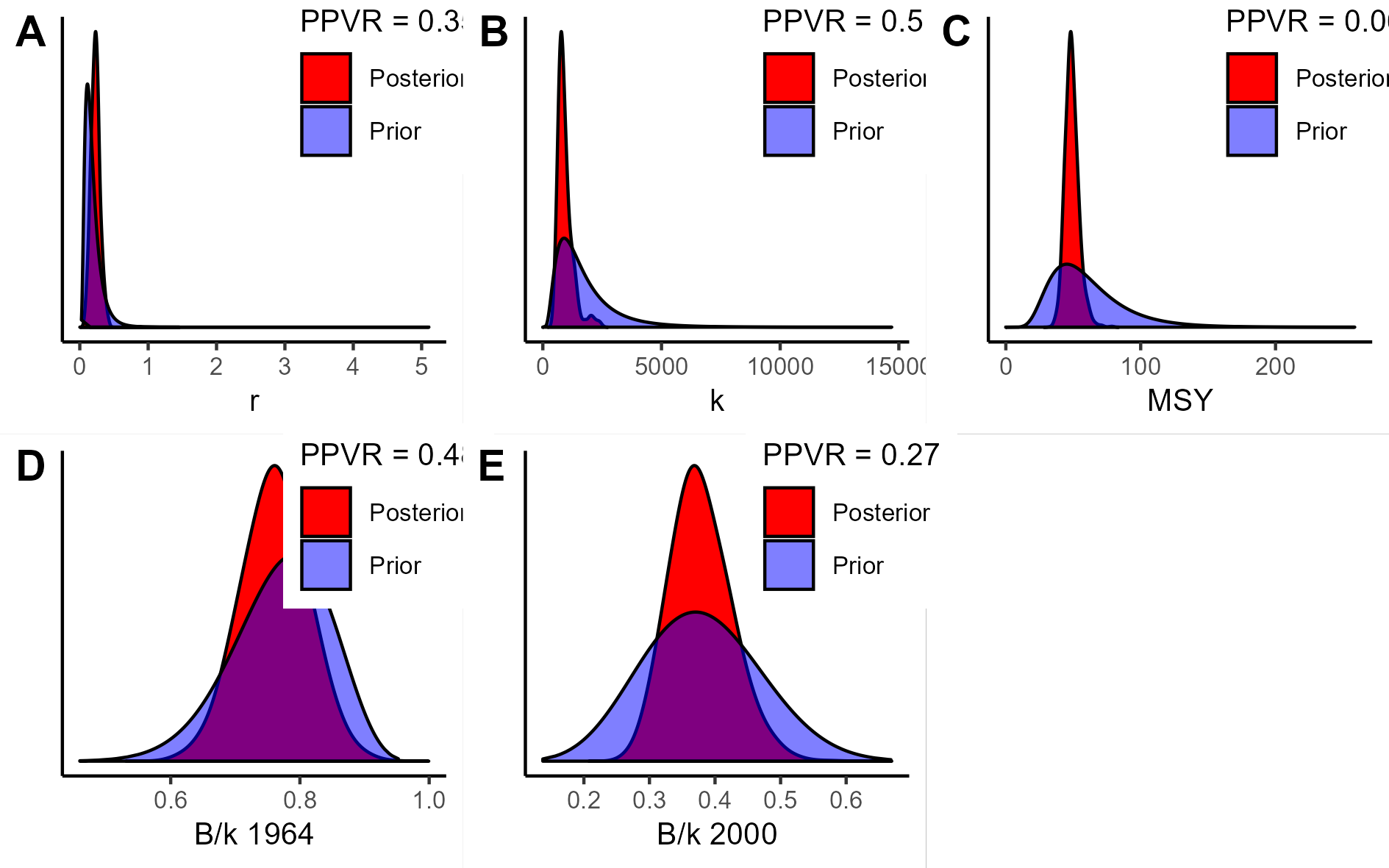

Supplement: S3 Fig — PPVR gives the ratio of posterior to prior variance. (TIFF) [file pone.0347768.s003.tiff]

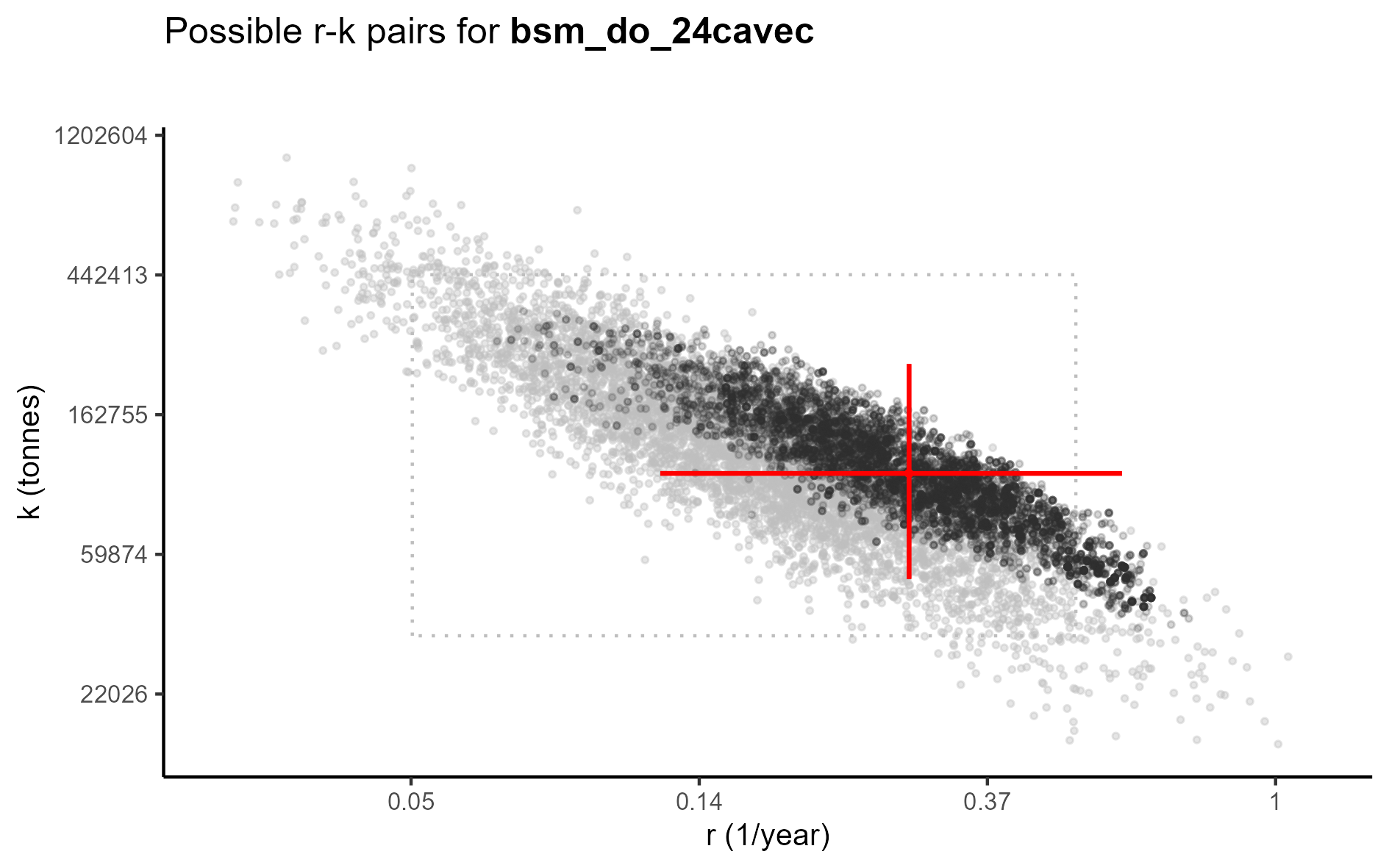

Supplement: S4 Fig — Black dots are viable r-k pairs found to be compatible with catch and CPUE data and the priors, with the most probable r-k pair indicted by the red cross (with CI). The dotted rectangle indicates the prior r-k range. (TIFF) [file pone.0347768.s004.tiff]

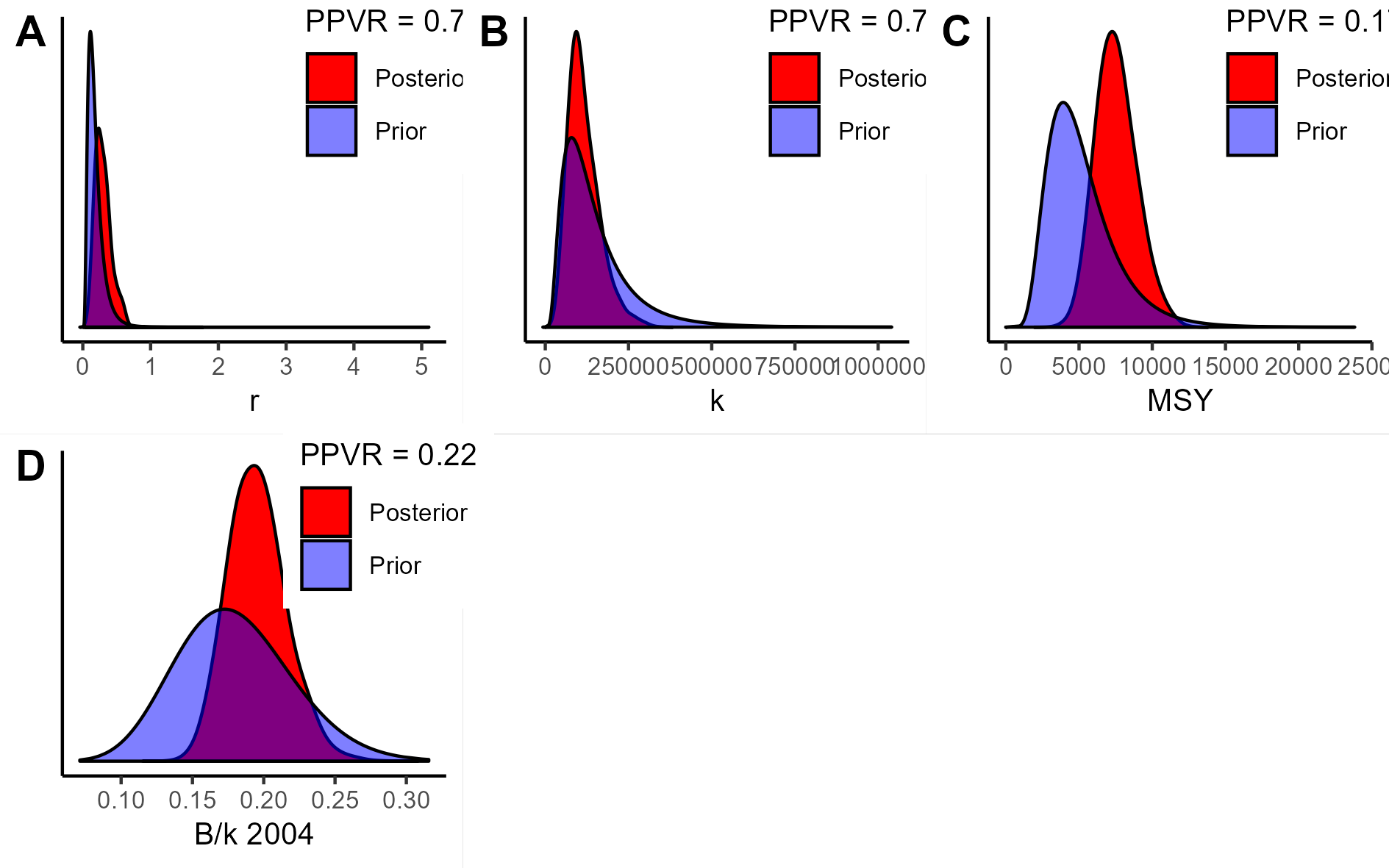

Supplement: S5 Fig — PPVR gives the ratio of posterior to prior variance. (TIFF) [file pone.0347768.s005.tiff]

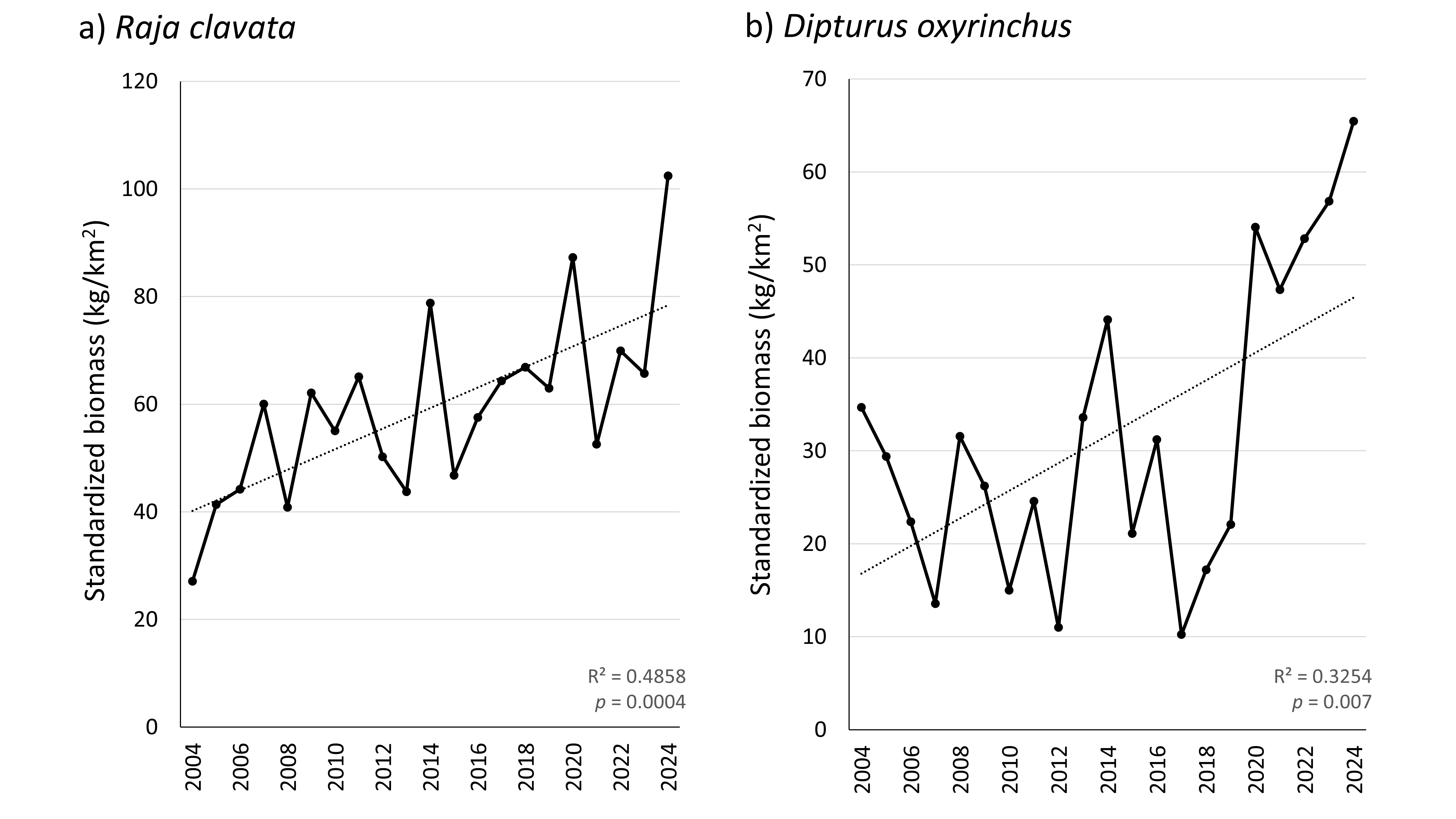

Supplement: S6 Fig — Linear regression lines (dotted) are shown with their coefficient of determination (R²) and p-values (p) for each species. (TIF) [file pone.0347768.s006.tif]
